# Profiling, clinicopathological correlation and functional validation of specific long non-coding RNAs for hepatocellular carcinoma

**DOI:** 10.1186/s12943-017-0733-5

**Published:** 2017-10-23

**Authors:** Jian Yao, Lingjiao Wu, Xiaohua Meng, Huanxia Yang, Shujun Ni, Qiangfeng Wang, Jiawei Zhou, Qiong Zhang, Kunkai Su, Li Shao, Qingyi Cao, Mingding Li, Fusheng Wu, Lanjuan Li

**Affiliations:** 1 State Key Laboratory for Dianosis and Treatment of Infectious Diseases, Collaborate Innovation Center for Diagnosis and Treatment of Infectious Diseases, The First Affiliated Hospital, College of Medicine, Zhejiang University, Hangzhou, 310003 China; 2 Department of Ultrasonography, The Women’s Hospital, School of Medicine, Zhejiang University, Hangzhou, 310006 China; 3 Department of Medical Oncology, Cancer Center, The First Affiliated Hospital, College of Medicine, Zhejiang University, Hangzhou, 310003 China; 4 Department of Surgical Oncology, the First Affiliated Hospital, College of Medicine, Zhejiang University, No. 79, Qingchun Rd, Hangzhou, 310003 China

**Keywords:** Hepatocellular carcinoma, Long non-coding RNA, RNA sequencing, Co-expression network, Cell migration

## Abstract

**Background:**

Hepatocellular carcinoma (HCC) is one of the most prevalent and aggressive malignancies worldwide. Studies seeking to advance the overall understanding of lncRNA profiling in HCC remain rare.

**Methods:**

The transcriptomic profiling of 12 HCC tissues and paired adjacent normal tissues was determined using high-throughput RNA sequencing. Fifty differentially expressed mRNAs (DEGs) and lncRNAs (DELs) were validated in 21 paired HCC tissues via quantitative real-time PCR. The correlation between the expression of DELs and various clinicopathological characteristics was analyzed using Student’s *t*-test or linear regression. Co-expression networks between DEGs and DELs were constructed through Pearson correlation co-efficient and enrichment analysis. Validation of DELs’ functions including proliferation and migration was performed via loss-of-function RNAi assays.

**Results:**

In this study, we identified 439 DEGs and 214 DELs, respectively, in HCC. Furthermore, we revealed that multiple DELs, including NONHSAT003823, NONHSAT056213, NONHSAT015386 and especially NONHSAT122051, were remarkably correlated with tumor cell differentiation, portal vein tumor thrombosis, and serum or tissue alpha fetoprotein levels. In addition, the co-expression network analysis between DEGs and DELs showed that DELs were involved with metabolic, cell cycle, chemical carcinogenesis, and complement and coagulation cascade-related pathways. The silencing of the endogenous level of NONHSAT122051 or NONHSAT003826 could significantly attenuate the mobility of both SK-HEP-1 and SMMC-7721 HCC cells.

**Conclusion:**

These findings not only add knowledge to the understanding of genome-wide transcriptional evaluation of HCC but also provide promising targets for the future diagnosis and treatment of HCC.

## Background

Hepatocellular carcinoma (HCC) is one of the most prevalent and aggressive malignancies worldwide [1], with incidence rates continuing to increase rapidly [2]. As a multistage progress, HCC typically arises from cirrhotic livers and is usually associated with viral hepatitis (hepatitis B and C viruses), foodstuffs contaminated with aflatoxin B1 (AFB_1_), alcohol abuse, and obesity [3]. Hepatocarcinogenesis is a complex progress, the initiation, promotion and progression of which is often involved with an accumulation of genetic and epigenetic changes [4]. In spite of the discovery and progression of the underlying molecular mechanisms of HCC, the current death rates continue to increase [2, 5].

Recently, growing evidence has suggested that long non-coding RNAs (lncRNAs), defined as transcripts larger than 200 nucleotides in length without evident protein coding functions, are capable of influencing multiple biological process through various mechanisms as guides, scaffolds, and decoys [6–8]. Moreover, reports have revealed that lncRNAs also have played a key role in gene regulation in cancer, affecting various aspects including proliferation, survival, migration or genomic stability [9–11]. Especially in HCC, several lncRNAs including highly up-regulated in liver cancer (HULC) [12], high expression in HCC (HEIH) [13], lncRNA associated with microvascular invasion in HCC (MVIH) [14], lncRNA metastasis-associated lung adenocarcinoma transcript 1 (MALAT-1) [15], maternally expressed gene-3 (MEG3) [16] and H19 [17], are usually deregulated and act as important regulators of cancer progression, providing potential biomarkers and candidate therapeutic targets for HCC diagnosis and treatment. Nevertheless, knowledge on the systematic profiling and characterization of HCC transcriptome (lncRNAs in particular) remains far from adequate.

In this study, with the application of RNA high-throughput sequencing (RNA-seq), we sequenced the transcriptome of 24 matched tissue samples (primary tumor and paired adjacent normal tissues) from 12 Chinese HCC patients, and validated the differentially expressed genes (DEGs) and lncRNAs (DELs) in 21 pairs of HCC tissue samples using quantitative real-time PCR (qRT-PCR). Next, we analyzed the association between DELs and clinicopathological characteristics, and found several lncRNAs that might serve as biomarkers of diagnosis and prognosis in HCC patients. Furthermore, the co-expression network between DEGs and DELs was applied to predict the potential functional and regulatory mechanisms of lncRNAs in HCC, and the possible functions of lncRNAs on HCC cells were experimentally validated.

## Findings

### Patients’ information and tissue collection

Tissue samples of fresh-frozen human primary hepatocellular carcinoma (HCC) and paired adjacent normal liver tissues (3 cm away from tumor) were collected from 24 Chinese patients undergoing surgery from October 2012 to October 2013 at the Department of Surgical Oncology, the First Affiliated Hospital, School of Medicine, Zhejiang University, China. The liver tissues were immediately snap-frozen in liquid nitrogen after surgery, and stored at −80 °C until use. The diagnosis of HCC was histologically confirmed via pathological examinations by two independent pathologists, and all the HCC tissues were assessed by hematoxylin and eosin (H&E) staining. Patients had no preoperative treatments. Twelve randomly selected paired samples (RNA-seq Set) were used for RNA sequencing as a discovery cohort, and 21 paired samples (Real-Time PCR Set, 9 were included in the RNA-seq Set) were used for qRT-PCR as a validation cohort. Clinicopathological features, including age, gender, HBV infection, tumor size, differentiation grade, serum α-fetoprotein (AFP) concentration, tissue immunohistochemical (IHC) staining status of AFP, and portal vein tumor thrombosis (PVTT) are summarized in Table 1.

**Table 1:** Clinicopathological characteristics of the patients included in this study

	RNA-seq Set	Real-Time PCR Set	Total
Median Age at Surgery (Range)	55(35–70)	55(35–74)	56(35–74)
≤50	5	9	9
>50	7	12	15
Sex			
Male	8	18	18
Female	4	3	6
HBsAg(+/−)			
–	2	3	4
+	10	18	20
Serum AFP (ng/ml)			
≤20	6	11	12
>20	6	10	12
Tumor Size (cm)			
≤5	10	14	17
>5	2	7	7
Tumor Differentiation			
Well/Moderate	7	11	13
Poor	5	10	11
PVTT			
–	8	15	17
+	4	6	7
IHC staining of AFP			
–	4	9	10
+	8	12	14
Total Number of patients	12	21^a^	24

^a^9 patients in the RNA-seq Set were included. *HBsAg* hepatitis B surface antigen, *AFP* alpha-fetoprotein, *PVTT* portal vein tumor thrombosis, *IHC* immunohistochemistry

### RNA-seq profiling of 12 HCC samples with paired adjacent normal liver tissues

To systematically identify the transcriptome of HCC, we performed RNA-seq on 12 HCC samples with paired adjacent normal liver tissues using Illumina Hiseq2500. Approximately 5.4 billion paired raw reads and 5.2 billion paired clean reads were generated for all 12 patients (average 45.2 million raw reads and 43 million clean reads each). After alignment, an average of 60 million reads mapped to human genome were acquired for each paired sample, accounting for 69.9% of the total clean reads. Specifically, the total of mapped annotated genes or long-noncoding RNAs (lncRNAs) was 53.6 million and 35.5 million (62.4% and 41.7%), respectively, for each patient.

### Identification of differentially expressed genes in HCC

After aligning all the reads to the human genome reference consortium GRCh37, we identified a total of 31,237 transcripts, including 29,427 transcripts in HCC tissues (T) and 29,280 in the paired adjacent normal tissues (N). Unsupervised Principal Components Analysis (PCA) of mRNAs demonstrated that HCC tissue samples can be distinctively separated from adjacent normal tissue samples.

**Fig. 1:**
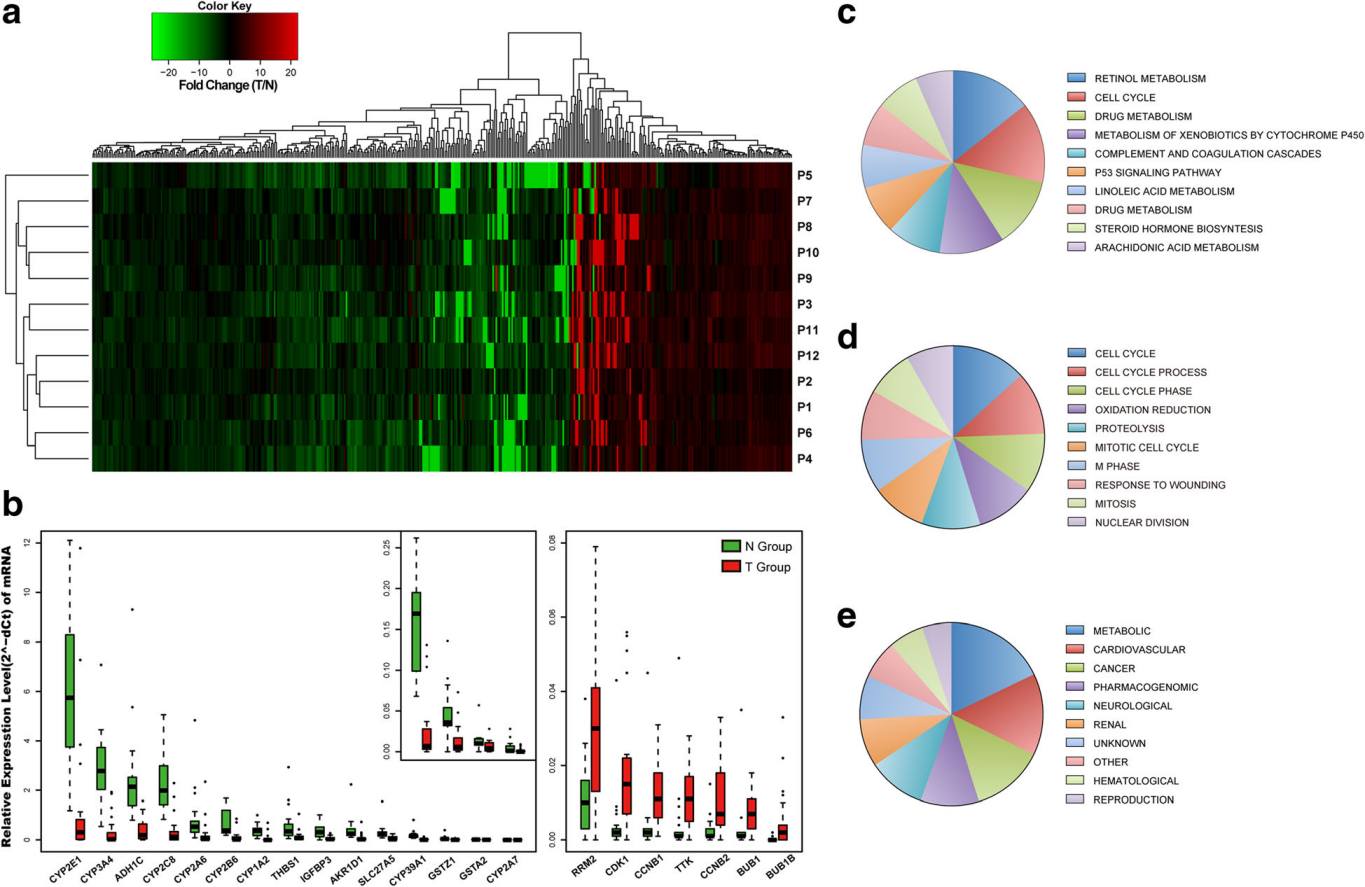
Expression differences and enrichment analysis of mRNAs in HCC tumor vs. paired adjacent normal tissues. **a** Hierarchical clustering analysis of 439 differentially expressed genes (DEGs) between 12 HCC tumor and paired adjacent normal tissue samples (log2-fold change >2 or <−2 and *P* < 0.05). Columns represent each gene, and rows represent each patient. The relative expression levels of mRNAs are depicted into color scale ranging from green (down-regulation) to red (up-regulation). **b** Real-time PCR validation of 25 DEGs in 21 HCC tumor and paired adjacent normal tissue samples, and 22 DEGs of statistical significance are presented in a box diagram (*P* < 0.05) with median bar indicated inside. Glyceraldehyde-3-phosphate dehydrogenase (GAPDH) is used as a reference gene. **c** Pie charts of the top ten KEGG pathways of 439 DEGs enriched by DAVID. **d** Pie charts of top ten GO terms for biological process. **e** Pie charts of the top ten terms for genetic associated diseases

Furthermore, we identified as many as 439 statistically significant differentially expressed genes (DEGs), the fold change of which was greater than 2 or less than 1/2 between T and paired N in at least half of the samples sequenced. Generally, there were 139 up-regulated and 300 down-regulated DEGs in HCC. The heat map of DEGs via hierarchical clustering analysis was generated and is displayed in Fig. 1a. Particularly, we noticed that only one gene named CENPF was up-regulated in all 12 pairs, while 12 genes including CFP, FOS, MT1 family, and NAT2 were down-regulated in all samples.

To better understand the enrichment of DEGs, we applied the Database for Annotation, Visualization and Integrated Discovery (DAVID) [18, 19] to analyze the distribution of DEGs through KEGG pathways, Gene Oncology (GO) and Genetic associated Disease database. Among the top 10 enriched KEGG pathways, most were related to metabolism except for the cell cycle and p53 signaling pathway, which were closely associated with tumor growth and development (Fig. 1c). We randomly selected 25 DEGs (15 down-regulated and 10 up-regulated) among the top enriched KEGG pathway terms, and validated their expression in 21 paired HCC tissues via qRT-PCR. As displayed in Fig. 1b, 22 DEGs (88%) were significantly dysregulated, 15 down-regulated and 7 up-regulated, in HCC. For GO, most were cell cycle related in the top 10 enriched terms, as well as terms involving oxidation reduction, proteolysis and response to wounding (Fig. 1d). In those terms related to disease, the metabolic related gene set was the most enriched term, while cancer related terms ranked third (Fig. 1e).

### Identification of novel differentially expressed lncRNAs in HCC

**Fig. 2:**
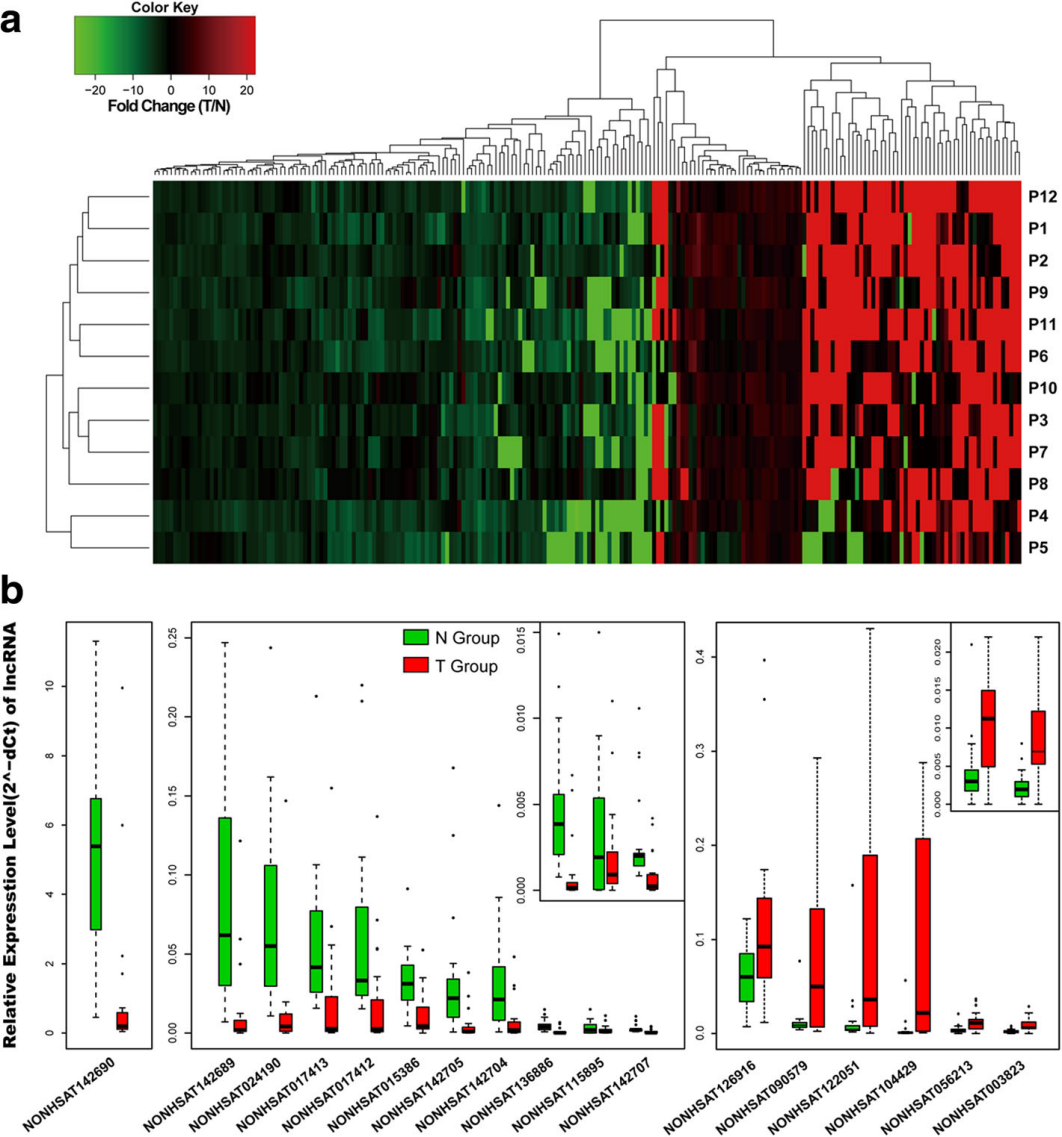
Expression differences of lncRNAs in HCC tumor vs. paired adjacent normal tissue samples. **a** Hierarchical clustering analysis of 214 differentially expressed lncRNAs (DELs) in HCC tumor samples. **b** Real-time PCR validation of 25 DELs in 21 HCC paired samples, and 17 of statistical significance are presented in box diagrams (*P* < 0.05). Beta-actin served as an internal control

By aligning the transcript reads to human ncrna_NONCODEv4 [20], we obtained a total of 71,106 (accounting for 77%) transcripts, including 65,302 in T and 65,433 in N. Similarly, by the same criteria as DEGs, we identified 214 differentially expressed lncRNA (DELs) transcripts in HCC, with 90 up-regulated and 104 down-regulated (Fig. 2a), much less than the number of DEGs (439). This finding is also consistent with previous reports showing that lncRNAs display higher expression variation than mRNAs [21]. We noted that the expression of four lncRNA transcripts, namely, NONHSAT001093, NONHSAT065819, NONHSAT142698, and NONHSAT142707, were down-regulated in all 12 patients, while the expression levels of NONHSAT056213, NONHSAT091576 and NONHSAT122271 were elevated in 10 out of 12 patients.

We randomly selected 25 DELs (16 down-regulated and 9 up-regulated), and verified their expression using qRT-PCR. As depicted in Fig. 2b, 17 DELs (68%) displayed significant dysregulation, 11 of which were down-regulated and 6 of which were up-regulated in HCC tissues. Meanwhile, we cross-referenced the expression profiles of these 25 DELs in TCGA database containing 200 HCC tissue samples and 50 normal control samples (TCGA Liver hepatocellular carcinoma, LIHC) from TANRIC [22], and found that 16 out of 17 DELs were consistent with our qRT-PCR results.

### Clinical relevance of novel DELs in HCC

**Fig. 3:**
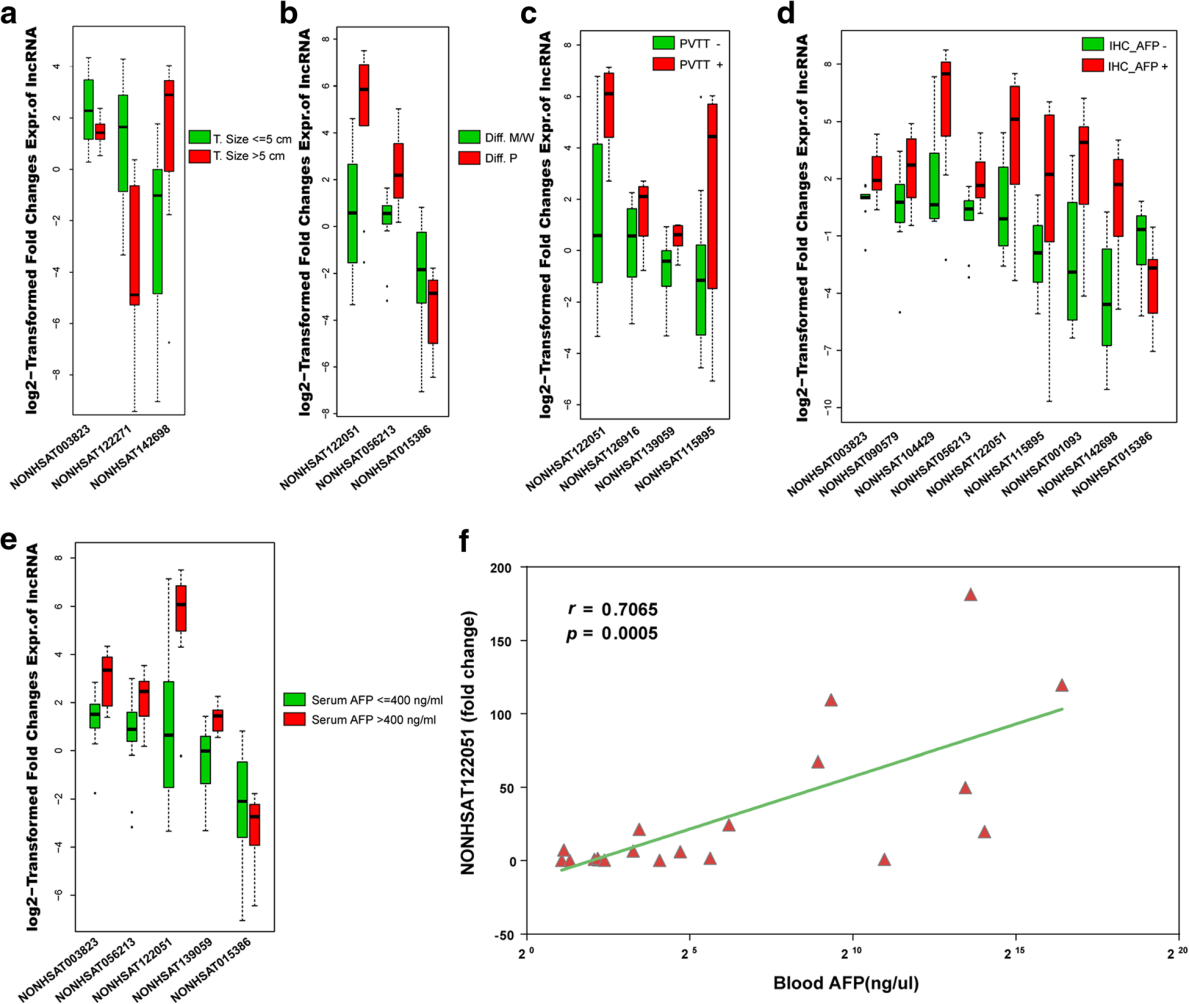
Correlation analysis of DELs with clinicopathologic characteristics of HCC. **a** The relative expression of DELs in 14 HCC patients with tumor size no larger than 5 cm vs. 7 patients with tumor size greater than 5 cm. The expression levels of DELs are normalized by the paired adjacent normal samples as fold changes and log2 transformed (*P* < 0.05, similarly hereinafter). **b** The relative expression of DELs in 11 patients with tumor histologic differentiation grade of well (W) or moderate (M) vs 10 with poor (P) differentiation grade. **c** The relative expression of DELs in 15 patients without portal vein tumor thrombosis (PVTT) and 6 patients with PVTT. **d** The relative expression of DELs in 9 HCC patients with negative immunohistochemistry staining of alpha-fetoprotein (AFP) in tumor tissues and 12 with positive staining. **e** The relative expression of DELs in 14 HCC patients with serum AFP level no more than 400 ng/ml, and 7 with more than 400 ng/ml. **f** The linear analysis of the relative expression of NONHSAT122051 and serum AFP concentration (*r* = 0.7065, *P* = 0.0005)

To further explore the association between DELs and clinicopathological characteristics of the HCC patients, we analyzed the expression profiles of 25 selective DELs based on the results of qRT-PCR. As presented in Fig. 3a, the expression levels of NONHSAT003823, NONHSAT122271 and NONHSAT142698 were significantly correlated with tumor size. Regarding tumor histologic differentiation, NONHSAT122051 and NONHSAT056213 were associated with poor differentiation, while NONHSAT015386 was up-regulated in well/ moderate differentiation grade (Fig. 3b). In contrast, NONHSAT122051, NONHSAT126916, NONHSAT139059 and NONHSAT115895 were positively correlated with portal vein tumor thrombosis (PVTT, Fig. 3c). It is of note that the expression levels of NONHSAT003823, NONHSAT056213, NONHSAT122051 and NONHSAT015386 were remarkably correlated with both the serum concentration and the tissue immunohistochemical (IHC) staining of α-fetoprotein (AFP) (Fig. 3d, e). Specifically, the expression level of NONHSAT122051 was linearly correlated with the serum AFP concentration with the *p* value of 0.0005 (Fig. 3f), suggesting that NONHSAT122051 may serve as a novel biomarker candidate for HCC.

### Co-expression network of DEGs and DELs in HCC

Because the biological functions of lncRNAs are largely unknown, we sought to explore potential functions by analyzing their co-expressed mRNA network. By analyzing the correlation between 439 DEGs and 214 DELs, we constructed a co-expression network containing a total of 2961 lncRNA-mRNA pairs, the absolute *r* value of which is above 0.8. Among these pairs, 262 were located on the same chromosome (*cis*-acting), accounting for only 8.8%. While most of these pairs (2698, 91.2%) were on the different chromosome (*trans*-acting), suggesting that most of the regulation of lncRNA and mRNA expression was in a *trans* way. These findings are also consistent with previous reports [23, 24].

**Fig. 4:**
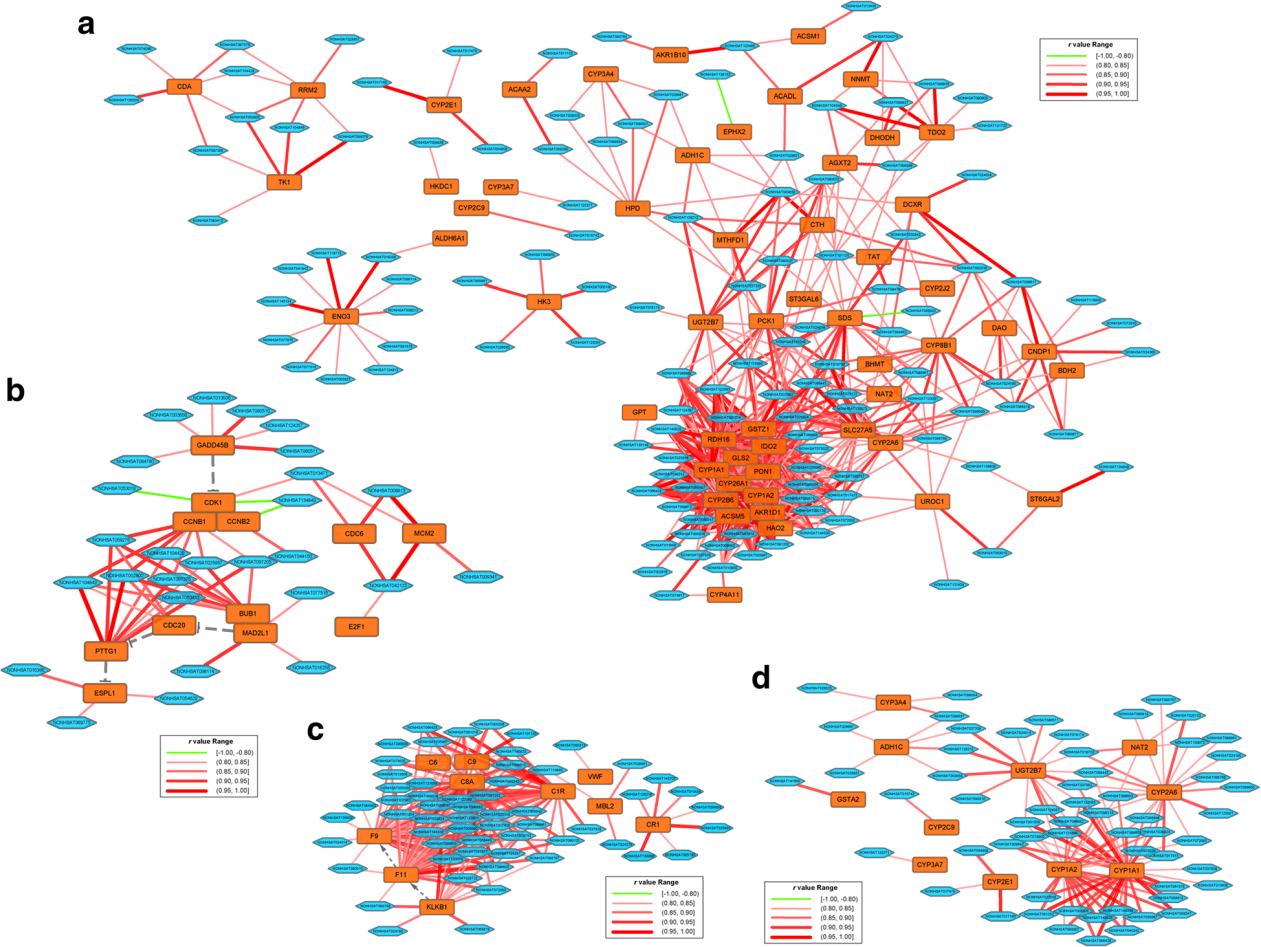
The co-expression network between DEGs and DELs in HCC. **a** The co-expression network of metabolic pathway containing 626 connections between 119 DELs and 55 DEGs with absolute *r* value larger than 0.8. The orange boxes represent DEGs, and the blue hexagons represent the DELs. The edges between DEGs and DELs indicate the potential correlation, of which the color and the thickness standing for different correlation coefficient were marked within the frame. **b** The co-expression network of cell cycle pathway containing 52 connections between 28 DELs and 12 DEGs. **c** The co-expression network of chemical carcinogenesis pathway containing 113 connections between 59 DELs and 11 DEGs. **d** The co-expression network of complementary and coagulation cascades pathway containing 179 connections between 58 DELs and 10 DEGs

Of the 2961 pairs, there were only 195 and 373 unique lncRNAs or mRNAs, respectively. Most of the lncRNAs (167, 85.6%) were differentially co-expressed with more than one mRNA. Likewise, 308 mRNA (82.6%) displayed a differential relationship with more than one lncRNA. Only 28 lncRNAs and 65 mRNAs showed single differential co-expression, suggesting that lncRNAs and mRNAs may tend to interact with each other in a complex network. Similar to the results of total DEGs enrichment, most of the enriched pathways involving the differentially co-expressed DEGs were metabolism related. The top enriched pathway, namely, metabolic pathways, contained 626 co-expression pairs, involving 55 DEGs and 119 DELs (Fig. 4a). It is noteworthy that cell cycle, chemical carcinogenesis, and complement and coagulation cascade-related pathways were listed within the top 10, containing 28, 59, and 58 DELs, respectively (Fig. 4b-d.), suggesting that these DELs may also play an important role during tumorigenesis and malignant progression.

### Functional validation of novel DELs in HCC

**Fig. 5:**
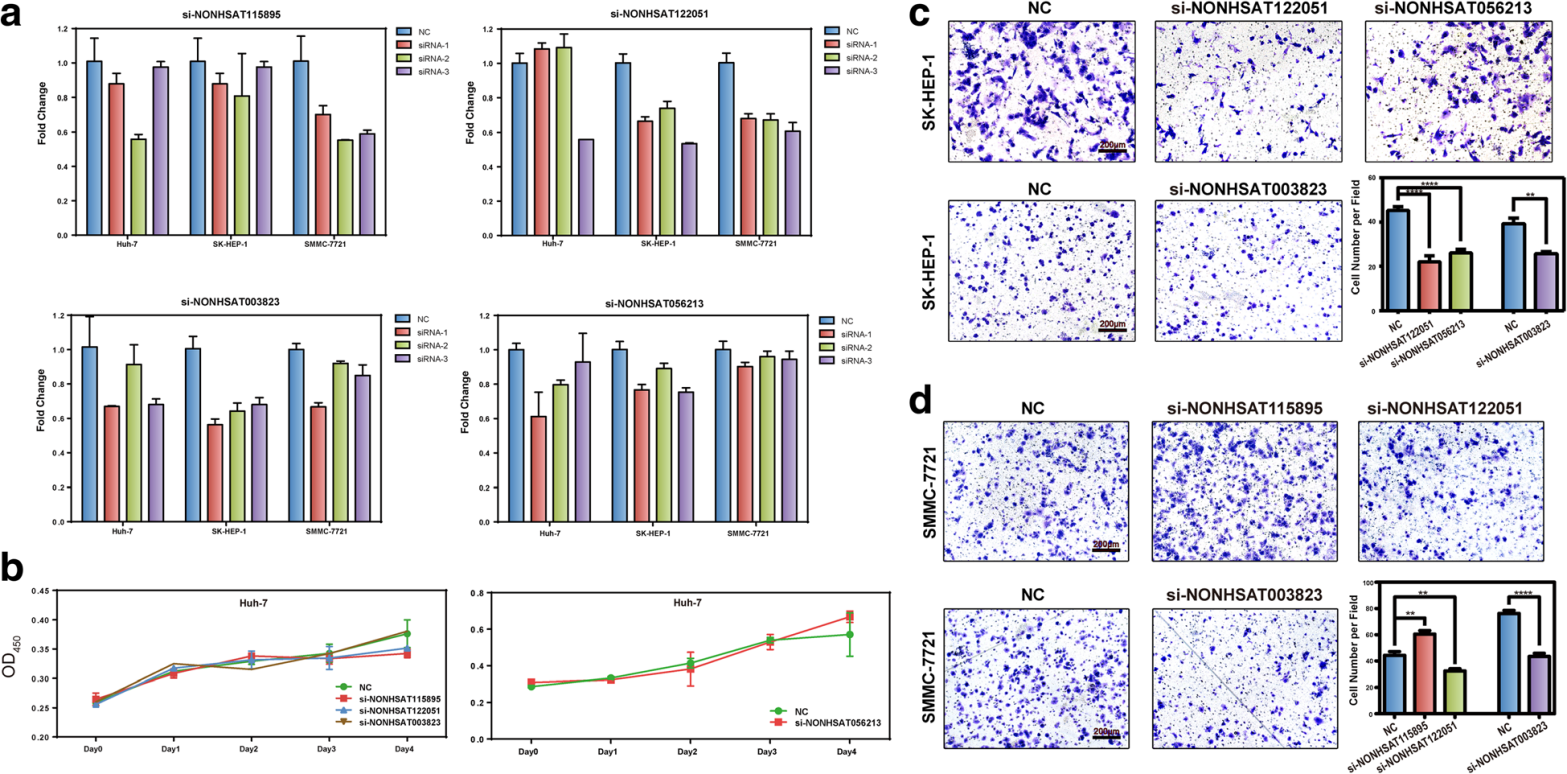
RNAi assay of candidate DELs regulating HCC cell proliferation and migration. **a** The relative expression levels of NONHSAT15985, NONHSAT122051, NONHSAT003823, and NONHSAT056213 in Huh-7, SK-HEP-1 or SMMC-7721 cells transfected with siRNAs or negative control (NC). **b** CCK-8 assay of Huh-7 cells transfected with siRNAs of NONHSAT115895, NONHSAT122051, NONHSAT003823, NONHSAT056213, or negative control (NC). Mean values are plotted as shown, and bars indicate SEM in triplet. **c** Transwell migration assays of SK-HEP-1 cells transfected with negative control (NC) or siRNAs for NONHSAT122051, NONHSAT056213, or NONHSAT003823. Representative images are shown, along with the quantification of five randomly selected fields. The values are depicted as the mean ± SEM; asterisks indicate significance. **d** Transwell migration assays of SMMC-7721 cells transfected with NC or siRNAs for NONHSAT115895, NONHSAT122051, or NONHSAT003823. Transwell assays were performed in triplicate, and the representative images are displayed

To investigate the potential biological functions of the DELs in HCC, loss-of-function assays were used by silencing the endogenous lncRNA levels in HCC cell lines via small interfering RNAs (siRNAs). Based on the knock-down effects (Fig. 5a), we transfected siRNA with the best efficiency into the HCC cell lines for each DEL. Despite little effect on the HCC cell proliferation (Fig. 5b), we did notice that they may have an impact on the cell migration ability. As shown in Fig. 5c, d, silencing the expression of NONHSAT122051 or NONHSAT003826 significantly attenuated the mobility of both SK-HEP-1 and SMMC-7721 HCC cells, indicating that they might play a potential pro-metastasis role in HCC development. In contrast, the knockdown of NONHSAT115895 remarkably promoted the migration of SMMC-7721, but the difference was of no statistical significance in SK-HEP-1, possibly due to the inefficient knock down (data not shown). Similarly, NONHSAT560213 could promote the migration of SK-HEP-1 cells, but no such significant difference was observed in SMMC-7721 (data not shown).

In recent transcriptomic studies, increasing evidence that long non-coding RNAs play a key regulatory role in gene regulation and chromatin remodeling has attracted more research attention. Despite accumulating evidence that lncRNA played an important role in cancers, the research on lncRNAs’ clinicopathological correlation with cancer, especially in HCC, are still far from adequate. It has been reported that the expression levels of CCAT1 [25], H19 [26], MEG3 [26], PANDAR [27], MALAT1 [28] and SPX [29] were significantly associated with serum AFP levels, while the expression levels of SPRY4-IT1 [30], SNHG1 [31], HNF1A-AS1 [32], HOTAIR [33], DDX5 [34] and GAS5 [35] were correlated with tumor differentiation. Moreover, GAS5, as well as SNHG3 [36], was also correlated with PVTT. A recent study using RNA-seq focused on the deregulated lncRNAs in HCC, especially with PVTT, identified multiple candidate lncRNAs for HCC tumorigenesis and metastasis, but failed to distinguish PVTT-specific lncRNAs from HCC tumor tissues [37]. In this study, we found that the up-regulation of NONHSAT056213, NONHSAT139059 and NONHSAT122051, as well as the down-regulation of NONHSAT015386 in HCC were positively correlated with the HCC clinicopathological features including poor tumor differentiation, PVTT, tissue or serum AFP levels. It is interesting to note that NONHSAT001093, NONHSAT115895 and NONSHAT142698 were down-regulated in HCC, but negatively correlated with tumor size, PVTT or AFP levels. Moreover, the expression of NONHSAT003823 was positively correlated with tissue or serum AFP levels, but negatively correlated with tumor size. The detailed biological functions and molecular regulation mechanisms of these DELs require further exploration.

Although research has indicated that lncRNAs are fine-tuners and key regulators in various aspects of cell transformation and metastasis especially in cancer, the exact functions of lncRNAs are not well understood [38–40]. To further functionally characterize lncRNAs, the analysis strategy named Guilt-By-Association (GBA) [41–43], which was originally derived from the strategy used for the data analysis of microarrays to identify gene sets sharing the similar features, was applied to construct a co-expression network of DELs and DEGs [44]. In our study, NONHSAT028824 were differentially co-expressed with 60 DEGs, most of which were metabolism related. We noticed that by genomic location there is a 588 bp overlap between NONHSAT028824 and apolipoprotein F (APOF), which may explain the high co-expression profiling (*r* = 0.9873). APOF is the protein component of apolipoproteins highly expressed restrictedly in liver [45], the aberrant expression of which would result in abnormality of lipid metabolism [46, 47], but its role in carcinogenesis remains unknown. In contrast, 3 out of 7 DEGs with the largest number of co-expressed DELs are members of complementary and coagulation cascades pathway (F11, C8A and C3P1). Recent reports indicate that the complement cascade facilitates oncogenesis and metastasis by sustaining proliferation, preventing apoptosis, promoting angiogenesis, invasion and migration, and suppressing antitumor immunity (reviewed in [48]). Similarly, the coagulation system can be activated by cancer cells and plays a role in tumor progression [49]. Our findings provide a novel thought on functional annotation of lncRNAs for future downstream experimental validation in HCC.

It is of particular note that NONHSAT122051 possibly serves as a novel promising biomarker for HCC. The loss-of-function experiment also demonstrated that NONHSAT122051 could significantly promote HCC cell migration. We noticed that the chromosome location of NONHSAT122051 overlaps with the coding gene paternally expressed 10 (PEG10), which could promote proliferation, migration and invasion in multiple cancer cells [50–52]. Furthermore, the abnormal expression of PEG10 is detected in various cancers [50, 53–56] including hepatocellular carcinoma [57]. It has been reported that the expression of lncRNAs is strikingly cell type-specific in normal tissues [58] and more cancer type-specific than protein coding genes [59]. Additionally, the relatively stable secondary structures of lncRNAs facilitates their detection as free RNAs, making them an ideal class of biomarkers for early detection, diagnosis and prognosis of cancers [9].

Due to the lack of sufficient tissue samples of HCC patients, we were unable to validate our findings in a larger cohort. The expression of about one-third (8, 32%) of DELs had no statistical significance between tumor and normal tissue samples. Nevertheless, resorting to the TCGA database in TANRIC, we found that a total of 21 DELs out of 25 displayed significantly differential expression in HCC, including 5 DELs failed in our qRT-PCR validation.

## Conclusion

Taken together, we identified a number of DEGs and DELs in HCC and further analyzed the clinicopathological correlation, as well as the potential functions of DELs. Our current findings may shed light on the overall understanding of HCC-specific lncRNAs, providing novel targets for the diagnosis and treatment of HCC.

## Abbreviations

AFB1: Aflatoxin B1
AFP: Alpha-fetoprotein
APOF: Apolipoprotein F
DAVID: Database for Annotation, Visualization and Integrated Discovery
DEG: Differentially expressed mRNA
DEL: Differentially expressed lncRNA
GAPDH: Glyceraldehyde 3-phosphate dehydrogenase
GBA: Guilt-By-Association
GO: Gene Oncology
H&E: Hematoxylin and eosin
HBsAg: Hepatitis B surface antigen
HCC: Hepatocellular carcinoma
HEIH: High expression in HCC
HULC: Highly up-regulated in liver cancer
IHC: Immunohistochemical
KEGG: Kyoto Encyclopedia of Genes and Genomes
lncRNA: Long non-coding RNA
MALAT-1: Metastasis-associated lung adenocarcinoma transcript 1
MEG3: Maternally expressed gene-3
MVIH: LncRNA associated with microvascular invasion in HCC
PCA: Principal Components Analysis
PEG10: Paternally expressed 10
PVTT: Portal vein tumor thrombosis
qRT-PCR: Quantitative real-time polymerase chain reaction
RIN: RNA integrity number
RNA-seq: RNA high-throughput sequencing
siRNA: Small interfering RNAs
